# Efficiency of indocyanine green fluorescence assisted laparoscopic hepatectomy in patients with hepatocellular carcinoma

**DOI:** 10.12669/pjms.41.1.10626

**Published:** 2025-01

**Authors:** Wei Feng, Xiaoxiao Xiong, Qile Wang, Siying Chen, Yao Ma, Dongming Zhu

**Affiliations:** 1 Wei Feng Department of General Surgery, The First Affiliated Hospital of Soochow University, Suzhou, Jiangsu Province 215006, P.R. China. Department of Hepatobiliary and Pancreatic Surgery, Nanjing Drum Tower Hospital Group Suqian Hospital, Suqian, Jiangsu Province 223800, P.R. China; 2 Xiaoxiao Xiong Department of Hepatobiliary and Pancreatic Surgery, Nanjing Drum Tower Hospital Group Suqian Hospital, Suqian, Jiangsu Province 223800, P.R. China; 3 Qile Wang Department of Hepatobiliary and Pancreatic Surgery, Nanjing Drum Tower Hospital Group Suqian Hospital, Suqian, Jiangsu Province 223800, P.R. China; 4 Siying Chen Department of Hepatobiliary and Pancreatic Surgery, Nanjing Drum Tower Hospital Group Suqian Hospital, Suqian, Jiangsu Province 223800, P.R. China; 5 Yao Ma Department of Hepatobiliary and Pancreatic Surgery, Nanjing Drum Tower Hospital Group Suqian Hospital, Suqian, Jiangsu Province 223800, P.R. China; 6 Dongming Zhu Department of General Surgery, The First Affiliated Hospital of Soochow University, Suzhou, Jiangsu Province 215006, P.R. China

**Keywords:** Indocyanine green fluorescence, Laparoscopic hepatectomy, Hepatocellular carcinoma

## Abstract

**Objective:**

To analyze the efficacy of indocyanine green fluorescence (ICG-F)-assisted laparoscopic hepatectomy in patients with hepatocellular carcinoma (HCC).

**Methods:**

This retrospective study included 120 patients with HCC who underwent laparoscopic hepatectomy in The First Affiliated Hospital of Soochow University from February 2020 to November 2022. Among them, 58 patients underwent conventional laparoscopic surgery (laparoscopic group), and 62 patients underwent ICG-F assisted laparoscopic surgery (ICG-F group). The perioperative conditions, levels of inflammatory factors before and after the operation, levels of liver function indexes, and incidence of complications in the two groups were statistically analyzed.

**Results:**

The perioperative indexes of patients in the ICG-F group improved compared to the laparoscopic group (*P*<0.05). On the second day after the operation, the inflammatory response in the two groups was significantly higher than before the operation and significantly lower in the ICG-F group compared to the laparoscopic group (*P*<0.05). Liver function of the two groups decreased two days after the surgery and was markedly lower in the ICG-F group than the laparoscopic group (*P*<0.05). The incidence of complications in the ICG-F group was significantly lower than that in the laparoscopic group (*P*<0.05).

**Conclusions:**

Compared with conventional laparoscopic surgery, ICG-F-assisted laparoscopic hepatectomy for HCC can reduce surgical injury, alleviate the degree of inflammatory response, protect patients’ liver function, and significantly reduce the risk of complications.

## INTRODUCTION

Hepatocellular carcinoma (HCC) is a type of multiple primary liver cancer that accounts for about 90% of the primary liver malignancies.^1^ The incidence of HCC is associated with hepatitis C virus infection, chronic hepatitis B, genetics, and other factors.^1^,^2^ The disease is characterized by a high metastasis and recurrence rate, as well as poor prognosis.^2^,^3^ Surgery remains an important treatment modality for the clinical treatment of HCC.^4^ While minimally invasive laparoscopic surgery can reduce surgical trauma and improve the efficacy and safety of treatment,^4^,^5^ it is associated with certain difficulties in tumor localization and liver segment labeling.^5^,^6^

With the continuous improvement of laparoscopic hepatectomy technology, indocyanine green fluorescence (ICG-F) navigation technology has gradually gained popularity.^6^,^7^ This method uses an intravenous injection of ICG, a water-soluble tri carbocyanine dye, for fluorescence staining of liver surface and liver parenchyma,^7^,^8^ and can provide clear and definite operation field for the surgical team and reduce the difficulty of the operation.^6^-^8^

However, due to the limitations of ICG-F technology, such as lack of tumor specificity and low penetration depth, its safety and effectiveness in laparoscopic hepatectomy remain controversial.^9^-^11^ Therefore, the purpose of this study was to retrospectively analyze clinical data of patients who underwent HCC resection in our hospital to clarify the application value of ICG-F assisted laparoscopic surgery and offer more evidence for the research of ICG-F-assisted laparoscopic surgery.

## METHODS

This retrospective study included patients with HCC who underwent laparoscopic hepatectomy in The First Affiliated Hospital of Soochow University from February 2020 to November 2022. Patients who underwent conventional laparoscopic surgery were categorized as the laparoscopic group, and patients who received ICG-F-assisted laparoscopic surgery were categorized as the ICG-F group.

### Inclusion criteria:

- Patients diagnosed with HCC^12^ and underwent laparoscopic hepatectomy.
- No distant metastases.
- Patient with Child-Pugh Class A or B.
- Complete clinical data.

### Exclusion criteria:

Patients with other malignant tumors.

- Patients with other important organ organic lesions.
- Patients with a history of drug allergy including allergy to ICG or iodine.
- Patients with extrahepatic metastases.
- Patients converted from laparoscopic hepatectomy to open hepatectomy.
- Patients with a history of abdominal surgery.

### Ethical Approval:

The ethics committee of The First Affiliated Hospital of Soochow University approved this study with the number 2017026, Date: November 10, 2017.

### Surgical procedures:

### Laparoscopic surgery:

The patient was placed in the supine position, and general anesthesia was administered. The incision was made at about one cm below the umbilicus. The endoscope was inserted to generate the carbon dioxide artificial pneumoperitoneum, and the pressure was controlled at 15 mmHg. The location of the lesion was confirmed by endoscopy, and the operation range was determined according to the exploration results. The accessory ligaments of the liver were resected by an ultrasonic scalpel, and liver lesions were completely exposed under laparoscopy. Resection was performed from about two cm around the lesion. After the operation, the drainage tube was placed, and the incision was closed.

### ICG-F assisted laparoscopic surgery:

**Supplementary Fig.1:**
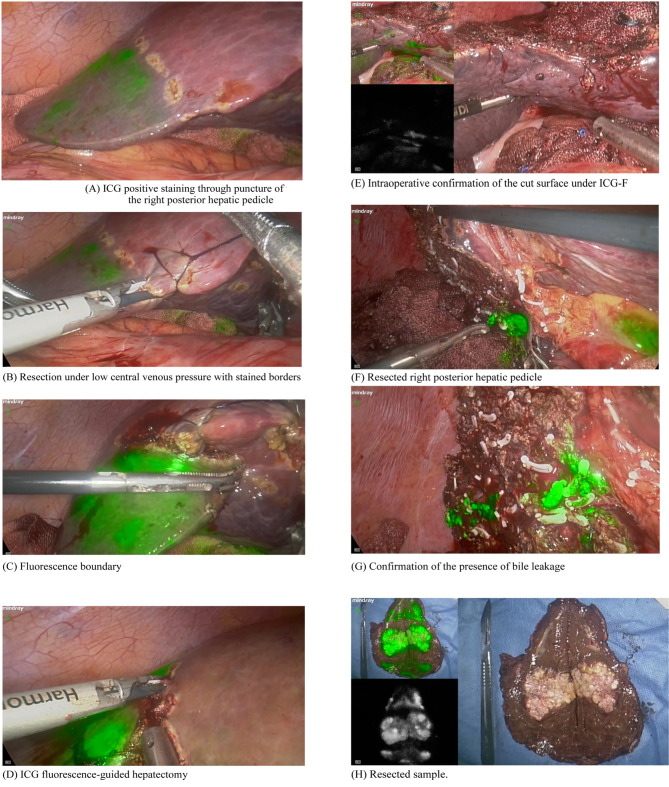
ICG-F assisted laparoscopic hepatectomy. (A) ICG positive staining through puncture of the right posterior hepatic pedicle; (B) Resection under low central venous pressure with stained borders; (C) Fluorescence boundary; (D) ICG fluorescence-guided hepatectomy; (E) Intraoperative confirmation of the cut surface; (F) Resected right posterior hepatic pedicle; (G) Confirmation of the presenc e of bile leakage; (H) Resected sample.

The patient was given a peripheral intravenous injection of 0.25 mg/kg ICG one day before the operation. The patient was placed in the supine position, and general anesthesia was administered. During the operation, the lesions were accurately located using the fluorescence and were locally resected along the fluorescence boundary under laparoscopy. The surgical margin had to be >1 cm from the tumor border. For deeper lesions, initial positioning was performed with ultrasound, and the state of the resection margin and the location of the tumor was observed in real-time using fluorescence during the resection process. The primary procedures are shown in Supplementary Fig.1.

### Outcome measures:

- Baseline data of patients, including gender, age, lesion diameter, and Child-Pugh classification of liver function.
- Perioperative conditions, including operation duration, intraoperative blood loss, drainage tube removal time, and length of hospital stay.
- Inflammatory factors, including levels of C-reactive protein (CRP), procalcitonin (PCT), and white blood cell count (WBC). A fully automated immunofluorescence analyzer (Roche, Cobas e8000, Switzerland) was used to detect serum CRP and PCT levels. A fully automated blood cell analyzer (Sysmex XN-10, Japan) was used for WBC.
- Liver function indexes, including levels of direct bilirubin (DBIL), total bilirubin (TBIL), aspartate aminotransferase (AST), and alanine aminotransferase (ALT). The levels of DBIL, TBIL, AST, ALT and other indicators were measured using a fully automated biochemical analyzer (Beckman Coulter, model AU5800, USA).
- Incidence of complications, including bile leakage, ascites, infection, pleural effusion, and fever.

### Statistical Analysis:

All data were analyzed using SPSS 25.0 software (IBM Corp, Armonk, NY, USA). The measured data were expressed as mean ± standard deviation. The students’ t-test was used to compare the groups. A paired t-test was used to compare the results before and after the treatment. The counting data were represented by the number of cases, using the chi-square test for comparison between groups. When *P*<0.05, the difference was considered statistically significant.

## RESULTS

A total of 120 patients (69 males and 51 females) who met the inclusion criteria were included. The mean age of the patients was 61.53 ± 7.14 years (range, 44-79 years). Of them, 58 patients underwent traditional laparoscopic surgery (laparoscopic group), and 62 underwent ICG-F assisted laparoscopic surgery (ICG-F group). There was no significant difference in baseline data between the two groups (*P*>0.05) (Table-I).

**Table-I:** Comparison of baseline data between the two groups.

Group	Gender (Male/Female)	Age (Year)	Lesion diameter (CM)	Child-Pugh Class (A/B)
ICG-F group (n=62)	39/23	62.12±6.96	3.79±1.38	41/21
Laparoscopic group (n=58)	30/28	60.97±7.31	3.83±1.41	31/27
*χ^2^/t*	1.532	0.883	-0.154	2.008
*P*	0.216	0.379	0.878	0.156

Operation time, drainage tube removal time, and hospitalization time of the ICG-F group were significantly shorter compared to the laparoscopic group, and the volume of intraoperative blood loss was significantly lower than that of the laparoscopic group (P<0.05) (Table II).

**Table-II:** Comparison of perioperative conditions between the two groups.

Group	n	Operation duration (minute)	Drainage tube removal time (day)	Length of stay (day)	Intraoperative blood loss (ml)
ICG-F group	62	141.63±21.84	4.29±1.13	10.63±1.96	101.72±19.06
Laparoscopic group	58	158.56±30.14	5.19±1.26	12.97±2.13	127.64±22.53
*t*		3.540	4.124	6.267	6.819
*P*		0.001	<0.001	<0.001	<0.001

Before the operation, there was no significant difference in the levels of CRP, PCT, and WBC between the two groups (*P*>0.05). On the 2^nd^ day after the operation, levels of CRP, PCT, and WBC in the two groups were significantly higher than those before the operation but significantly lower in the ICG-F group compared to the laparoscopic group (P<0.05) (Table III).

**Table-III:** Comparison of inflammatory factor levels between the two groups.

Time	Group	n	CRP (mg/L)	PCT (ng/L)	WBC (×10^9^/L)
Before operation	ICG-F group	62	8.13±1.29	0.61±0.16	8.29±1.52
	Laparoscopic group	58	8.32±1.35	0.59±0.19	8.51±1.39
	*t*		0.788	0.625	0.826
	*P*		0.432	0.533	0.411
On the 2^nd^ day after operation	ICG-F gropu	62	12.56±2.14	0.86±0.18	12.49±1.72
	Laparoscopic group	58	14.98±2.35	0.99±0.20	15.01±2.79
	*t*		5.904	3.747	5.998
	*P*		<0.001	<0.001	<0.001

***Note:*** compared with the group before operation, ^a^P<0.05.

There was no significant intergroup difference in the preoperative levels of DBIL, TBIL, AST, and ALT (*P*>0.05). On the 2^nd^ day after the operation, these indexes were significantly lower than before the operation and were markedly lower in the ICG-F group compared to the laparoscopic group (*P*<0.05) (Table-IV). The incidence of complications in the ICG-F group (4.84%) was significantly lower compared to the laparoscopic group (17.24%) (*P*<0.05) (Table-V).

**Table-IV:** Comparison of liver function indexes between the two groups.

Time	Group	n	DBIL (umol/L)	TBIL (umol/L)	AST (U/L)	ALT (U/L)
Before operation	ICG-F group	62	34.50±5.19	44.13±8.26	76.65±14.42	62.15±9.40
	Laparoscopic group	58	35.31±4.91	42.96±9.39	79.01±11.77	60.64±10.31
	*t*		0.877	0.726	0.978	0.839
	*P*		0.382	0.469	0.330	0.403
On the 2^nd^ day after operation	ICG-F group	62	10.26±2.35	30.62±5.17	36.68±10.26	36.65±8.19
	Laparoscopic group	58	14.38±3.69	36.65±7.21	48.50±13.35	45.60±7.32
	*t*		7.344	5.291	5.459	6.296
	*P*		<0.001	<0.001	<0.001	<0.001

***Note:*** compared with the group before operation, ^a^P<0.05.

**Table-V:** Comparison of the incidence of complications between the two groups.

Group	n	Bile leakage	Ascites	Infection	Pleural effusion	Fever	Overall incidence
ICG-F group	62	1 (1.61)	1 (1.61)	0 (0.00)	0 (0.00)	1 (1.61)	3 (4.84)
Laparoscopic group	58	3 (5.17)	2 (3.45)	1 (1.72)	2 (3.45)	2 (3.45)	10 (17.24)
*χ* ^2^							4.772
*P*							0.029

## DISCUSSION

This study shows that ICG-F-assisted laparoscopic hepatectomy for HCC patients is more efficient in reducing surgical trauma and is associated with a significantly lower risk of complications compared with conventional laparoscopic surgery. Our results are generally consistent with previous research.^10^,^11^,^13^ Our results suggest that ICG-F-assisted laparoscopic hepatectomy is beneficial for ensuring the early recovery of postoperative body function. In addition, the ICG-FI technology can achieve precise positioning of tumors, allow more accurate assessment of cancer foci during the surgery, and effectively shorten surgery time.

Recent studies have demonstrated the effectiveness of ICG-F imaging in detecting primary HCC, extrahepatic metastases, and liver blood flow.^14^-^18^ ICG-F binds to plasma protein after intravenous injection and will remain in the diseased tissue when the liver function is impaired. Felli E et al.^19^ pointed out that ICG-F-assisted laparoscopic surgery can improve the effectiveness and safety of hepatectomy. Urade T et al.^20^ showed that ICG-F-assisted laparoscopic hepatectomy can improve the visualization of lesion boundaries and the identification of hepatectomy boundaries. Moreover, it improves the accuracy of tumor lesion resection, helps reduce surgical trauma, and reduces the risk of postoperative complications. Lieto E et al.^2^ confirmed that ICG-F can improve the accuracy of staging assessment of primary and metastatic liver tumors. Zhou Y et al.^21^ showed that ICG-F-assisted laparoscopic surgery was associated with shorter operation time and postoperative hospitalization time, reduced intraoperative blood loss, and reduced risk of complications. These reports are consistent with our results. Taken together, these results indicate that ICG-F can improve the accuracy of tumor location, reduce the difficulty of surgical operation, and facilitate rapid and effective tumor resection by the surgical team.^2^,^19^-^21^

Identifying the exact location and boundary of tumor lesions in conventional laparoscopic surgery is complicated, which makes it difficult to effectively protect liver parenchyma due to positive resection margin, narrow resection margin, and other factors, which may lead to the postoperative decline in liver function.^22^ The results of our study show that ICG-F-assisted laparoscopic surgery effectively protects liver function in patients with HCC. We may speculate that using ICG-F during the laparoscopic surgery allows the operating surgeon to monitor the cutting edge in real time and to adjust the pre-tangent continuously. This, in turn, contributes to improved accuracy of surgical resection, reduces damage to normal liver tissue, and ensures early recovery of postoperative liver function, consistent with previous reports.^22^-^25^ Berardi G et al.^26^ pointed out that ICG-F can accurately locate the boundary of tumor lesions, obtain satisfactory surgical margins, ensure accuracy and safety of the surgery, detect small volume and deep lesion tissues, improve surgical resection rate, and alleviate the adverse effects of residual tumor lesions.

Although laparoscopic surgery has the advantage of minimal invasiveness, it can still result in varying degrees of trauma, leading to liver/systemic inflammatory responses, which, in turn, may promote the occurrence and progression of the tumor and metastases.^27^ The results of this study show that ICG-F-assisted HCC resection can reduce the degree of inflammatory response in patients. It is plausible that this effect of ICG-F is due to improved accuracy of surgical treatment and effective protection of liver parenchyma, which reduce the damage and alleviate the degree of inflammatory response.^27^,^28^

### Limitation:

This is a retrospective analysis, and the sample size is small, which may lead to selection bias. In addition, the impact of ICG-F assisted laparoscopy on long-term functional recovery and patient prognosis was not analyzed. Further multi-center large-scale research with longer follow-up time is needed to verify our results.

## CONCLUSION

Compared with conventional laparoscopic surgery, ICG-F-assisted laparoscopic resection can reduce surgical trauma, lower the degree of inflammatory response, protect liver function, and significantly reduce the risk of complications in patients with HCC.

## Authors’ contributions:

**WF** was contributed to the study design and manuscript writing.

**XX, QW, SC, YM and DZ** were involved in data collection, data analysis and interpretation.

**WF** was involved in the manuscript revision and validation and is responsible for the integrity of the study.

All authors have read and approved the final manuscript.
